# Pulsed Facilitation of Corticospinal Excitability by the Sensorimotor μ-Alpha Rhythm

**DOI:** 10.1523/JNEUROSCI.1730-19.2019

**Published:** 2019-12-11

**Authors:** Til Ole Bergmann, Anne Lieb, Christoph Zrenner, Ulf Ziemann

**Affiliations:** ^1^Department of Neurology and Stroke and Hertie Institute for Clinical Brain Research, Eberhard Karls University of Tübingen, 72076 Tübingen, Germany,; ^2^Institute for Medical Psychology and Behavioral Neurobiology, Eberhard Karls University of Tübingen, 72076 Tübingen, Germany, and; ^3^Deutsches Resilienz Zentrum, 55131 Mainz, Germany

**Keywords:** alpha oscillation, motor cortex, motor evoked potential (MEP), real-time EEG-TMS, short-interval intracortical inhibition (SICI), transcranial magnetic stimulation (TMS)

## Abstract

Alpha oscillations (8–14 Hz) are assumed to gate information flow in the brain by means of pulsed inhibition; that is, the phasic suppression of cortical excitability and information processing once per alpha cycle, resulting in stronger net suppression for larger alpha amplitudes due to the assumed amplitude asymmetry of the oscillation. While there is evidence for this hypothesis regarding occipital alpha oscillations, it is less clear for the central sensorimotor μ-alpha rhythm. Probing corticospinal excitability via transcranial magnetic stimulation (TMS) of the primary motor cortex and the measurement of motor evoked potentials (MEPs), we have previously demonstrated that corticospinal excitability is modulated by both amplitude and phase of the sensorimotor μ-alpha rhythm. However, the direction of this modulation, its proposed asymmetry, and its underlying mechanisms remained unclear. We therefore used real-time EEG-triggered single- and paired-pulse TMS in healthy humans of both sexes to assess corticospinal excitability and GABA-A-receptor mediated short-latency intracortical inhibition (SICI) at rest during spontaneous high amplitude μ-alpha waves at different phase angles (peaks, troughs, rising and falling flanks) and compared them to periods of low amplitude (desynchronized) μ-alpha. MEP amplitude was facilitated during troughs and rising flanks, but no phasic suppression was observed at any time, nor any modulation of SICI. These results are best compatible with sensorimotor μ-alpha reflecting asymmetric pulsed facilitation but not pulsed inhibition of motor cortical excitability. The asymmetric excitability with respect to rising and falling flanks of the μ-alpha cycle further reveals that voltage differences alone cannot explain the impact of phase.

**SIGNIFICANCE STATEMENT** The pulsed inhibition hypothesis, which assumes that alpha oscillations actively inhibit neuronal processing in a phasic manner, is highly influential and has substantially shaped our understanding of these oscillations. However, some of its basic assumptions, in particular its asymmetry and inhibitory nature, have rarely been tested directly. Here, we explicitly investigated the asymmetry of modulation and its direction for the human sensorimotor μ-alpha rhythm. We found clear evidence of pulsed facilitation, but not inhibition, in the human motor cortex, challenging the generalizability of the pulsed inhibition hypothesis and advising caution when interpreting sensorimotor μ-alpha changes in the sensorimotor system. This study also demonstrates how specific assumptions about the neurophysiological underpinnings of cortical oscillations can be experimentally tested noninvasively in humans.

## Introduction

Alpha (8–14 Hz) oscillations are the most prominent rhythm observable during wakefulness in the human scalp EEG (Berger, 1929). They are strongly expressed in all sensory regions (Haegens et al., 2015) and presumably involve both thalamic and cortical generators (Lopes da Silva et al., 1980; Vijayan and Kopell, 2012). According to the pulsed inhibition hypothesis (Klimesch et al., 2007; Jensen and Mazaheri, 2010), alpha cycles reflect bouts of inhibition, rhythmically suppressing bottom-up processing of sensory input, restricting associated gamma (40–100 Hz) oscillations (Tallon-Baudry and Bertrand, 1999) to interleaved periods of disinhibition. Importantly, alpha has been proposed to be asymmetric (Mazaheri and Jensen, 2008; Schalk, 2015), with larger amplitudes reflecting stronger inhibition and shortened periods of disinhibition, resulting in fewer gamma cycles and reduced information processing capacity (Jensen et al., 2014).

Indeed, alpha power and phase modulate gamma oscillations in human visual (Osipova et al., 2008) and motor cortex (Yanagisawa et al., 2012), and neural spiking in monkey motor and somatosensory cortex (Haegens et al., 2011). Also visual cortical excitability, indexed by perceptual performance or the probability of transcranial magnetic stimulation (TMS) to induce phosphenes has been inversely linked to occipital alpha power (Thut et al., 2006; Romei et al., 2008a,b; van Dijk et al., 2008) and is modulated by its phase (Busch et al., 2009; Mathewson et al., 2009; Dugué et al., 2011). Accordingly, transcranial alternating current stimulation (TACS) in the alpha range phasically suppressed visual stimulus-induced gamma power in concurrent MEG recordings, with the extent of phasic suppression predicting the accompanying decrease in visual detection performance (Herring et al., 2019).

**Figure 1.**
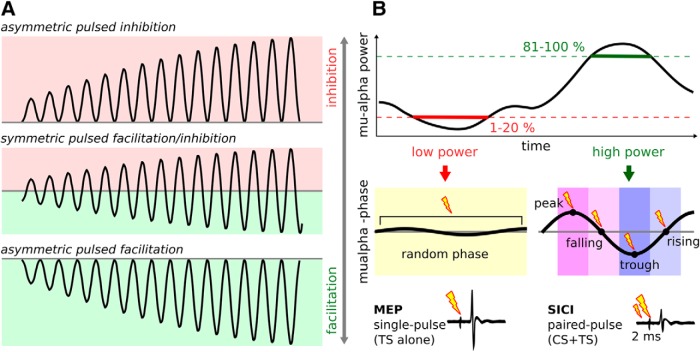
Scenarios for a rhythmic modulation of corticospinal excitability by the sensorimotor μ-alpha rhythm and illustration of detection criteria for real-time EEG-triggered TMS. ***A***, Three different possible scenarios of rhythmic modulation of corticospinal excitability by the sensorimotor μ-alpha oscillation: asymmetric pulsed inhibition, producing stronger inhibition with increasing amplitude as predicted by the 'pulsed inhibition hypothesis' (top); symmetric pulsed inhibition and facilitation, both stronger with increasing amplitude (middle); or asymmetric pulsed facilitation, producing stronger facilitation with increasing amplitude (bottom). ***B***, EEG-triggered single-pulse TMS (test stimulus, TS, alone to assess MEPs) and paired-pulse TMS (with preceding conditioning stimulus, CS+TS at 2 ms ISI to assess short-latency intracortical inhibition, SICI) targeting periods of low (1–20% percentile) and high (80–100% percentile) μ-alpha power. The low power condition was targeted at random phase, whereas for the high power condition, either peak (0°), falling flank (90°), trough (180°), or rising flank (270°) of the ongoing μ-alpha rhythm were targeted. TS, Test stimulus; CS, conditioning stimulus.

For the sensorimotor μ-alpha rhythm, the link to cortical excitability is less consistent. In primary somatosensory cortex (S1), both negative linear (Jones et al., 2010; Anderson and Ding, 2011) and inverted u-shape relationships (Linkenkaer-Hansen et al., 2004; Zhang and Ding, 2010; Anderson and Ding, 2011; Ai and Ro, 2014) have been observed between prestimulus μ-alpha power and tactile perception or somatosensory evoked potentials. In the primary motor cortex (M1), earlier studies either observed negative relationships in small samples (Zarkowski et al., 2006; Lepage et al., 2008; Sauseng et al., 2009), or no relationship at all (for review, see Madsen et al., 2019), whereas more recent studies suggest a positive linear relationship with motor evoked potential (MEP) amplitude (Hussain et al., 2018; Thies et al., 2018; Ogata et al., 2019). Our group previously observed μ-alpha phase to modulate corticospinal excitability, with larger MEPs evoked during *troughs* compared with *peaks* of μ-alpha waves (Schaworonkow et al., 2018, 2019; Stefanou et al., 2018; Zrenner et al., 2018). However, it remained unknown whether this phasic modulation reflects asymmetric pulsed inhibition, asymmetric pulsed facilitation, or a symmetric combination of both (Fig. 1*A*), and if cortical excitability depends on phase or merely the instantaneous voltage amplitude (Schalk, 2015). To answer these questions, we used real-time EEG-triggered single- and paired-pulse TMS to measure corticospinal excitability (MEP amplitude) and GABA-A-receptor mediated short-latency intracortical inhibition (SICI) (Kujirai et al., 1993) at rest (i.e., with relaxed muscles and in absence of any motor task) at four different phase angles (peak, falling flank, trough, rising flank) of a robustly expressed (i.e., high power) spontaneous μ-alpha rhythm and compared them to a baseline state of spontaneously desynchronized (i.e., low power) μ-alpha at random phase when the rhythm is virtually absent (Fig. 1*B*). If μ-alpha reflects asymmetric pulsed inhibition, then its less excitable peaks should reflect inhibition and attenuate MEPs relative to low power periods and troughs alike, possibly accompanied by a rhythmic increase of SICI. If μ-alpha reflects asymmetric pulsed facilitation instead, MEPs should be increased during troughs relative to low power periods and peaks, and no modulation of SICI should be observed. A symmetric scenario would result in some combination of the above. Further, if phase per se matters regardless of voltage amplitude, excitability may differ for rising and falling flanks despite comparable absolute voltages.

## Materials and Methods

### 

#### 

##### Subjects.

Twenty-three (*n* = 23) healthy, right-handed volunteers (26.1 ± 5.8 years; 11 females), who were free of medication and had no neurologic or psychiatric history or any contraindications against TMS (Rossi et al., 2011), participated after providing written informed consent. The study protocol conformed to the Declaration of Helsinki and was approved by the local ethics committee of the University Hospital Tübingen. Subjects were recruited based on the following inclusion criteria: (1) a clear μ-alpha frequency peak (i.e., a distinct peak between 8 and 14 Hz in the power spectrum with an amplitude ≥2× the background 1/f noise, as visually identified in the eyes-open EEG resting-state power spectrum; see below) to ensure sufficient signal-to-noise-ratio for real-time power and phase targeting; and (2) the existence of a TMS motor hot spot allowing to consistently evoke MEPs with a resting motor threshold (RMT) ≤75% maximum stimulator output (MSO) to ensure sufficiently long stimulation periods without coil overheating. In total, 23 of 36 screened subjects fulfilled these criteria, were included, and completed the study.

##### Procedures.

Subjects participated in a single session, consisting of several preparatory measures and the main experiment. Preparatory measures (see below for details) included: mounting of EEG and EMG electrodes, arrangements for TMS neuronavigation, EEG resting-state recording (3 min) for calibration of real-time detection criteria, motor hot spot search, as well as automated determination of resting motor threshold (RMT), stimulation intensity (SI) producing MEPs of 1 mV peak-to-peak amplitude, and CS intensity producing 50% of maximal SICI based on a SICI curve with varying CS intensities. During the main experiment, both single-pulse TMS (TS alone) and paired-pulse TMS (CS + TS at 2 ms interstimulus interval, ISI) was delivered, assessing corticospinal excitability and GABA-A-receptor mediated intracortical inhibition respectively. TMS was automatically triggered in real-time (see below for details) to target 5 different μ-alpha states: (1) low μ-alpha power periods (i.e., 1–20% of the individual μ-alpha power distribution) at random phase, or high μ-alpha power periods (i.e., 81–100% of the individual μ-alpha power distribution) at four different phase angles of the μ-alpha rhythm, i.e., either (2) the peak (0°), (3) the falling flank (90°), (4) the trough (180°), or the rising flank (270°). These 10 different experimental conditions (5 μ-alpha rhythm states × 2 trial types) were pseudorandomly intermingled (by concatenating permutations of the 10 conditions). The experiment was split into multiple blocks, separated by ∼10 min breaks to allow for coil cooling and relaxation time for the participant. To account for slow power drifts with time on task (Benwell et al., 2019), in the first 16 subjects, the break was also used to perform a recalibration of μ-alpha power thresholds (see below) based on 3 min resting-state EEG recordings, whereas in the last 7 subjects a continuous recalibration was implemented in form of a sliding distribution of μ-alpha power values based on the last 60 s of clean data (excluding 1.5 s intervals post-TMS), as this procedure had been shown in the meanwhile to prevent unnecessarily long intertrial intervals (ITI) that occur when the algorithm waits for the power criterion to be met in the face of slow μ-alpha power fluctuations (Thies et al., 2018). This resulted on average in slightly shorter and more homogenous ITIs for the last seven compared with the first 16 subjects (3.7 ± 0.7 s vs 4.6 ± 1.1 s), but did not produce any differences between experimental conditions. Block duration varied based on individual stimulation intensity (i.e., max. time until coil required cooling) and individual endogenous μ-alpha rhythm fluctuations (i.e., actual average ITI due to EEG-triggered TMS), resulting on average in 4.6 ± 1.1 blocks (M ± SD) with 15.2 ± 4.0 min duration and a total number of 95.3 ± 13.1 trials (min: 70, max 123) acquired per condition.

##### EEG recordings.

64-channel EEG via extra-flat TMS-compatible sintered Ag/AgCl electrodes (Multitrodes, EasyCap) and 2-channel EMG were recorded in DC mode with 1000 Hz anti-aliasing low-pass filter and digitized at 5 kHz using a TMS-compatible 24-bit amplifier (NeurOne Tesla with Digital-Out Option, Bittium). EMG was recorded from the relaxed right first dorsal interosseus (FDI) muscle in belly-tendon montage via a bipolar channel of the same amplifier.

##### TMS.

TMS was applied to the left M1 via four Magstim 200^2^ stimulators, connected to a single 70 mm figure-of-eight coil via the Magstim 4-into-1 module to allow paired-pulses with 2 ms ISI and ITI <4 s (recharge time of a single Magstim 200^2^ unit). Coil position was determined to produce consistent MEPs in the target muscle and was maintained using neuronavigation (Localite). Monophasic stimuli induced a posterolateral-to-anteromedial current in the brain tissue. Stimulation intensity (SI) for the suprathreshold test stimulus (TS) was set to elicit MEP amplitudes around 1 mV (SI1mV: 60.3 ± 10.8% MSO), and SI for the subthreshold conditioning stimulus (CS) 2.0 ms earlier was set to produce 50% of maximal possible SICI (31.5 ± 5.6% MSO or 65.6 ± 9.1% RMT) as determined from the SICI curve (see below) to allow a bidirectional modulation of SICI by the μ-alpha rhythm, while preventing floor or ceiling effects.

##### EEG resting-state recording.

Resting-state EEG was recorded for 3 min with subjects having their eyes open and fixating a crosshair in ∼2 m distance as well as keeping their muscles relaxed. Power spectra were calculated by a Hanning-windowed fast Fourier transform (FFT) for consecutive, nonoverlapping 1 s data segments, and individual μ-alpha frequency was determined as frequency bin of maximal power in the 8–14 Hz range of the 1/f corrected power spectrum. Further, individual power thresholds for low and high μ-alpha power conditions were determined as the 20% and 81% percentile, respectively, from the individual distribution of μ-alpha power values during the 3 min recording.

##### TMS threshold hunting.

Resting motor threshold (RMT) and stimulation intensity inducing ∼1 mV MEPs on average (SI1mV) were determined using a fully automated adaptation of the Simple Adaptive Parameter Estimation by Sequential Testing (SA-PEST) procedure (Taylor et al., 1983; Awiszus, 2003; Borckardt et al., 2006), which we implemented in MATLAB using our real-time EEG/EMG system to read out the MEP response to the last TMS pulse and adjust the SI for the next pulse accordingly to reach a fluctuating equilibrium with half of the MEPs being smaller or larger than the target value, respectively (i.e., 0.05 mV for RMT and 1 mV for SI1mV). After a fixed number of 40 trials, SI was averaged over the last 20 as an estimate of the respective threshold.

##### SICI curve.

SICI, calculated as ratio of the MEP evoked by CS + TS relative to the TS alone, was calculated for 10 different CS intensities (ranging from 45% to 90% MSO in steps of 5% with a fixed TS intensity at SI1mV) intermingled in pseudorandomized order with 20 trials per CS intensity and 20 trials of the TS alone. Based on the SICI curve interpolated from all these intensities, the CS intensity was then determined that caused ∼50% of the maximal possible inhibition in a given individual.

##### Real-time EEG-TMS.

**Figure 2.**
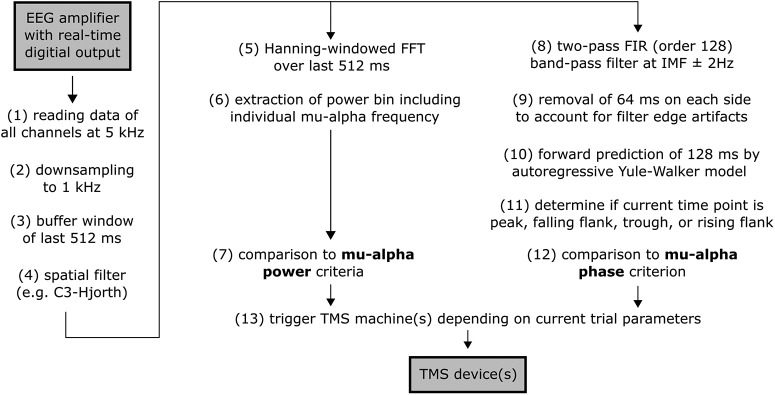
Overview of real-time EEG-triggered TMS processing pipeline. See Materials and Methods for details. IMF, Individual μ-alpha frequency.

The real-time EEG-TMS system is described previously in detail (Zrenner et al., 2018). Briefly, a Simulink Real-Time (R2016a; The MathWorks) model processed the EEG data at 1 kHz and triggered TMS whenever the respective power and phase criteria were met (see Fig. 2 for a schematic overview of the real-time processing pipeline). Real-time EEG processing involved: (1) reading in digitized data of 64 EEG- and 2 EMG-channels from the NeurOne system, (2) downsampling to 1 kHz, (3) buffering the last 512 ms data with a sliding window, (4) spatial filtering with a C3-centered Hjorth-montage [C3 − mean(CP1, CP5, FC1, FC5)] (Hjorth, 1975) to create a single virtual channel; and for power targeting: (5) calculating a Hanning-windowed FFT of the last 512 ms sliding data segment, (6) extracting the frequency bin including individual μ-alpha peak frequency (10.9 ± 1.1 Hz M±SD), (7) comparing the current μ-alpha power value to the power criteria targeted in the current trial (with power percentiles determined either from the resting-state calibration preceding the current run (first 16 subjects) or from a sliding distribution of μ-alpha power values (last 7 subjects), see details below); and for phase targeting: (8) band-pass filtering the last 512 ms sliding data segment of the raw C3-Hjorth signal by a two-pass (zero-phase) finite impulse response filter (FIR) filter with order 128 and a pass-band of the individual μ-alpha frequency ± 2 Hz, (9) removing the 64 ms corrupted by filter edge artifacts on each side of the buffer, (10) forward predicing the signal based on the remaining 384 ms by an autoregressive model (Yule–Walker, order 30) for 128 ms (McFarland and Wolpaw, 2008; Chen et al., 2013), thus providing ± 64 ms around “time 0” (i.e., “now”), (11) determining whether the data point at time 0 is a maximum turning point (i.e., a peak), a minimum turning point (i.e., a trough), a negative-to-positive zero crossing (i.e., a rising flank), or a positive-to-negative zero crossing (i.e., a falling flank), (12) comparing the current μ-alpha phase to the phase criterion targeted in the current trial; and eventually: (13) immediately triggering either a single or a paired TMS-pulse (depending on the current trial type) if both the current power and phase criteria are met for the data point at time 0. Scalp-to-Simulink data transmission delay was ∼ 3 ms (no jitter), and processing time per real-time cycle and TMS trigger delays accumulated to ∼ 1 ms; including a slight Simulink-to-MagStim trigger delay TMS thus was applied with an average delay of ∼4.5 ms. A minimal ITI of 3 s was maintained to avoid corruption of power or phase estimates by TMS-related brain responses or artifacts from the previous trial.

##### Offline EEG analysis.

*Post hoc* offline-analyses were only performed to validate detection performance of the real-time EEG analyses. Pre-TMS EEG data were processed offline, using the FieldTrip toolbox (Oostenveld et al., 2011) and custom MATLAB code (The MathWorks), to verify that TMS was correctly delivered to the intended μ-alpha states. EEG data were segmented (−1.5 to 1 s relative to TMS), baseline corrected (−0.502 to −0.002 s, avoiding the TMS pulse artifact), and rereferenced to the common average of all EEG electrodes. A virtual channel was added, representing the C3-centered Hjorth-montage [C3 − mean(CP1, CP5, FC1, FC5)](Hjorth, 1975). Independent component analysis (ICA) was conducted on pre-TMS data segments (−1.002 to −0.002), down-sampled to 1 kHz, to identify components reflecting eye movement artifacts and muscle noise based on their spatial topography, spectral profile, as well as their temporal profiles within and across trials (Chaumon et al., 2015). Subsequently, the same unmixing matrix was applied to the original data, previously identified bad components were removed (on average 2.1 ± 0.9 eye movement components and 3.7 ± 2.3 muscle components per subject), and data were projected back to channel space. Subsequently, semiautomatic artifact detection was used to reject trials with either EMG preinnervation (amplitude >50 μV in the 80–140 Hz band-pass filtered EMG signal) or EEG artifacts in C3-Hjorth (*z*-normalized signal > 5 SDs in the 1 Hz high-pass filtered EEG signal) in the pre-TMS period (on average 3.67 ± 1.51 trials per condition were rejected per subject). Although ITI (4.33 ± 1.08 s, mean ± SD) did not differ significantly at the group level, neither between phase conditions (*p* > 0.2) nor between single and paired-pulse trials (*p* > 0.7), conditions were stratified per subject with respect to ITI to exclude any possible confound of MEP amplitude by variations in ITI (Julkunen et al., 2012; Vaseghi et al., 2015). Single-subject stratification iteratively removed trials with the longest ITI from conditions with the longest average ITI until a repeated-measures ANOVA (rmANOVA) of ITI across conditions reached a *p-*value ≥ 0.2 (Thies et al., 2018). On average, 73.6 ± 1.9 trials remained per condition after bad trial rejection and stratification. To demonstrate power-specificity, power spectra were calculated per trial using a Hanning-windowed FFT of the pre-TMS interval (−0.502 to −0.002 s), zero-padded to 1 s, with a frequency resolution of 1 Hz, ranging from 1 to 35 Hz, and spectra were averaged per condition across trials and afterward across subjects. To show the frequency-specificity to the targeted μ-alpha power, time–frequency representations (TFR) were calculated for the pre-TMS, for a time period from −1.5 to 1 s, with the post-TMS period being replaced by zeros to prevent any TMS-related responses and artifacts of the post-TMS period from corrupting power estimates in the pre-TMS period. We applied Welch's method using a moving Hanning-windowed FFT with a dynamic window length of 3 cycles of a given frequency, a step size of 20 ms, and a frequency resolution of 1 Hz, ranging from 1 to 35 Hz. Since TMS was delivered in a μ-alpha power- and phase-triggered fashion, there was no unbiased baseline period preceding the TMS-pulses to allow commonly used normalization as relative change from baseline. TFRs for each subject were therefore *z*-normalized per condition with respect to the average across all conditions before calculating grand averages across subjects. To show topographical specificity of the targeted μ-alpha power, the topographical distribution of *z*-normalized pre-TMS μ-alpha power values (as extracted from the individual μ-alpha peak frequency bin and averaged across the −0.3 to −0.1 s time bins of the TFR) was plotted per condition. To illustrate phase-specificity, pre-TMS time-series were averaged across trials per subject and condition. Time-series were converted to phase-angle (in radians) according to the individual μ-alpha peak frequency before averaging across subjects to account for interindividual differences in μ-alpha frequency and prevent phase-cancelation when averaging across subjects. Average phase of TMS application was quantified per condition and subject. Since no detected target states were left unstimulated to maximize trial numbers, the phase at which TMS was actually applied could not be directly calculated due to signal corruption by TMS-related artifacts and -evoked potentials. As second best alternative, phase was thus estimated for the uncorrupted time point exactly one individual μ-alpha cycle earlier. To increase precision, individual μ-alpha period was not determined from the initial resting-state power spectrum, but from the main experiment as the average interpeak (and intertrough) interval from the last three μ-alpha cycles preceding the TMS pulse (10.8 ± 1.1 Hz M±SD; absolute deviation from initial peak frequency estimation was 0.4 ± 0.4 Hz). Phase estimates revealed a delay corresponding to ∼4.5 ms, attributable to technical factors (see Materials and Methods) which have been taken into account in newer versions of the real-time algorithm.

##### Offline EMG analysis.

MEP peak-to-peak amplitudes from all remaining trials (73.6 ± 1.9 per condition, see above) were normalized block-wise as percentage change from block average (across all conditions) and then averaged across blocks to take slow drifts in corticospinal excitability across blocks into account (Thies et al., 2018; Zrenner et al., 2018). SICI was calculated per μ-alpha rhythm state as ratio of the MEP evoked by paired-pulse TMS relative to single-pulse TMS, and was additionally normalized per subject as percentage of the maximal inducible SICI (from the SICI curve).

##### Experimental design and statistical analysis.

The experiment consisted of a single session per subject (*n* = 23, 11/12 female/male). The independent variable was the targeted μ-alpha state, realized as a within-subject factor with the following five power/phase combinations as levels: low/random, high/peak, high/rising, high/trough, and high/falling. The two dependent variables were corticospinal excitability as indexed by MEP amplitude and GABA-A-receptor mediated inhibition as indexed by the 2 ms SICI of MEP amplitudes. For both dependent variables, one-way rmANOVAs were conducted with *post hoc* paired *t* test where applicable. Statistical analyses were conducted using MATLAB (functions *RMAOV1* and *ttest*). *p* < 0.05 was considered significant. Effect sizes for ANOVA (η_p_^2^, partial η squared) and *t* tests (Cohen's *d*_av_, based on the averaged SD) are provided (Lakens, 2013). In addition, we report the Bayes factor, calculated using the JASP statistical software package (JASP Team, jasp-stats.org), for nonsignificant tests as BF_01_ to quantify strength of evidence supporting the null hypothesis (H0) and for significant tests as BF_10_ (i.e., 1/BF_01_) to quantify strength of evidence supporting the alternative hypothesis (H1). According to Jeffreys (1961), a Bayes factor of 1–3 reflects “anecdotal evidence”; 3–10, “substantial evidence”; 10–30, “strong evidence”; 30–100, “very strong evidence”; and >100, “decisive evidence” for the H0 (BF_01_) and H1 (BF_10_), respectively. Data are reported as mean ± SEM (M ± SEM) if not stated otherwise. EEG data were merely analyzed as a manipulation check; that is, to demonstrate successful μ-alpha power and phase targeting.

## Results

### μ-alpha rhythm phasically facilitates MEP corticospinal excitability but not intracortical inhibition

**Figure 3.**
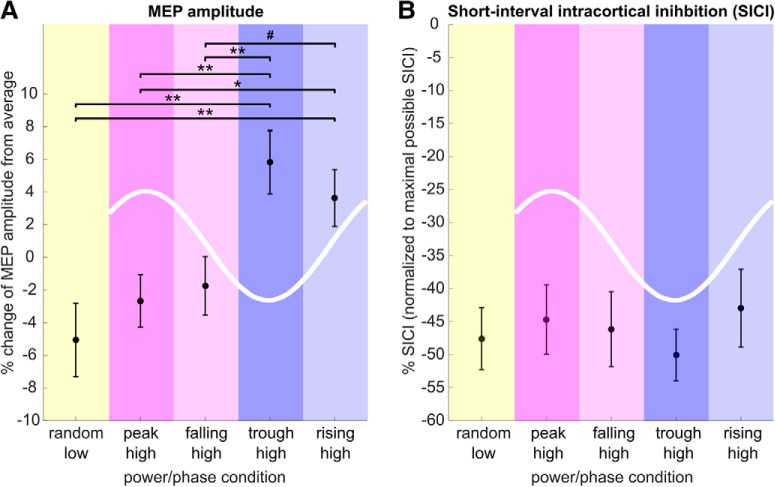
MEP amplitude but not SICI is modulated by power and phase of the sensorimotor μ-alpha rhythm. ***A***, Normalized MEP amplitude (percentage change from block average across conditions; mean ± 1 SEM) was modulated by both μ-alpha power and phase (*F*_(4,88)_ = 4.71, *p* = 0.002). Although MEPs for high-power peaks and falling flanks did not differ from the low-power random phase condition, MEPs during high-power troughs and rising flanks were significantly increased relative to both low-power trials and high-power peak and rising flank conditions. Significance of *post hoc* comparisons is indicated as follows: #*p* < 0.1; *p < 0.05; **p < 0.01. ***B***, Normalized SICI, or the ratio of conditioned to unconditioned MEP as percentage of individual maximum SICI (mean ± 1 SEM), is modulated neither by μ-alpha power nor by μ-alpha phase (all *p* > 0.3).

MEP amplitude was modulated as a function of μ-alpha power and phase (*F*_(4,88)_ = 4.71, *p* = 0.002, η_p_^2^ = 0.18, BF_10_ = 107.92; Fig. 3*A*, Table 1). When averaged across phase conditions, MEPs triggered during periods of high μ-alpha power were larger than those obtained at random phase during low μ-alpha power (t_22_ = 2.25, *p* = 0.03, Cohen's *d*_av_ = 0.80, BF_10_ = 1.76). Taking phase into account, MEPs were larger during the μ-alpha trough and rising flank than during the peak and falling flank (trough vs peak: t_22_ = 2.97, *p* = 0.008, *d*_av_ = 0.99, BF_10_ = 6.09; trough vs falling: t_22_ = 2.83, *p* = 0.009, *d*_av_ = 0.85, BF_10_ = 5.04; rising vs peak: t_22_ = 2.19, *p* = 0.04, *d*_av_ = 0.78, BF_10_ = 1.59; with a trend for rising vs falling: t_22_ = 1.8, *p* = 0.08, *d*_av_ = 0.64, BF_10_ = 0.88), but did not differ between trough and rising flank (*p* > 0.4, *d*_av_ = 0.25, BF_01_ = 3.37) or between peak and falling flank (*p* > 0.6, *d*_av_ = 0.11, BF_01_ = 4.26). Importantly, MEPs during high power trials were only increased with respect to low power trials when obtained during the trough and rising flank (trough: t_22_ = 2.94, *p* = 0.008, *d*_av_ = 1.08, BF_10_ = 6.18; rising: t_22_ = 2.25, *p* = 0.03, *d*_av_ = 0.90, BF_10_ = 6.71), but not during the peak and falling flank (peak: *p* > 0.4, *d*_av_ = 0.26, BF_01_ = 3.37; falling: *p* > 0.3, *d*_av_ = 0.34, BF_01_ = 3.04). In contrast, while being clearly expressed at all phase angles (Table 1), SICI did not differ significantly as a function of μ-alpha power or phase (*F*_(4,88)_ = 1.104, *p* = 0.36, η_p_^2^ = 0.05, BF_01_ = 7.076; Fig. 3*B*). MEP amplitudes were thus rhythmically facilitated during the trough and rising flank of high amplitude μ-alpha oscillations, while remaining comparable to periods of low μ-alpha amplitude during high amplitude peaks and falling flanks. This modulation was not mediated by variations in intracortical inhibition.

**Table 1.** Mean ± 1 SEM for MEP amplitudes and short-latency intracortical inhibition (SICI) per condition: raw MEP amplitudes, normalized MEP amplitudes, SICI, and normalized SICI

Power/Phase	Low/Random	High/Peak	High/Falling	High/Trough	High/Rising
MEP (mV raw)	1.26 ± 0.10	1.31 ± 0.11	1.33 ± 0.12	1.44 ± 0.13	1.39 ± 0.12
MEP (% normalized)	−5.06 ± 2.25	−2.65 ± 1.60	−1.74 ± 1.78	5.82 ± 1.94	3.63 ± 1.74
SICI (% of TS)	−35.28 ± 4.02	−33.23 ± 3.88	−35.10 ± 4.01	−36.39 ± 3.55	−32.26 ± 4.45
SICI (% of max. SICI)	−47.58 ± 4.72	−44.67 ± 5.27	−46.15 ± 5.68	−50.06 ± 3.90	−42.96 ± 5.89

### Real-time EEG-triggred TMS sucessfully targeted μ-alpha power and phase conditions

**Figure 4.**
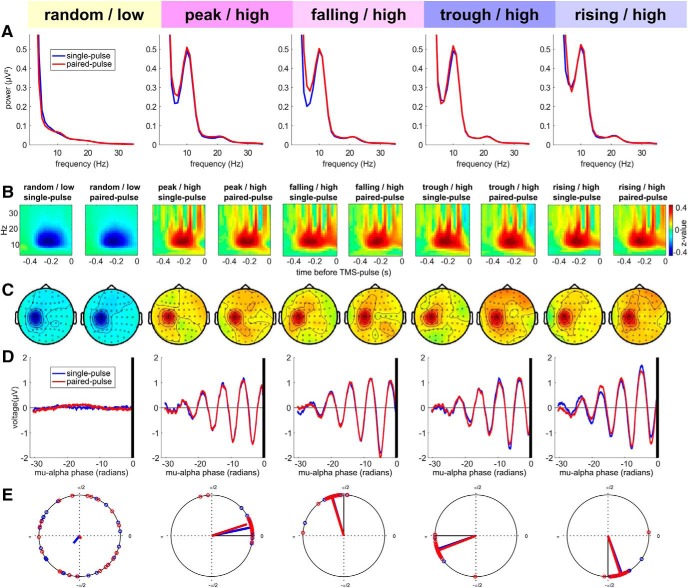
μ-alpha power- and phase conditions were successfully targeted. ***A***, Pre-TMS power spectra (FFT) of the C3-Hjorth signal for single- (blue) and paired-pulse trials (red), separately for all power/phase conditions. A clear μ-alpha peak can be observed in all high power conditions but not in the low power condition. ***B***, Pre-TMS TFRs of oscillatory power in the C3-Hjorth signal, calculated separately for single- and paired all power/phase conditions and *z*-normalized across conditions. TFRs show a modulation of μ-alpha power preceding TMS onset (at 0 ms), with a relative increase for high power trials and a relative decrease for low power trials. Note that the apparent broad-band bursts of oscillatory power (vertical bands) in the high power condition are explained by the fact that each of those trial types was time-locked to a specific phase of the nonsinusoidal μ-alpha oscillation. Also note that the apparent decrease in modulation shortly before TMS results from to zero-padding of the post-TMS interval to prevent corruption of pre-TMS interval by overlapping of the sliding window (length: three cycles per frequency) with TMS-related activity or artifacts. ***C***, Topographical maps of the *z*-normalized pre-TMS μ-alpha power modulation (time window [−0.3 −0.1] from ***B***). The topographies verify that a local power increase over left sensorimotor cortex was targeted, and estimates were not confounded by the stronger parieto-occipital alpha oscillation. ***D***, Time-locked C3-Hjorth signal relative to delivery of TMS (black vertical line) for single- (blue) and paired-pulse trials (red) with the time-axis transformed to phase angle (in radians) of the individual μ-alpha peak frequency before averaging across subjects to prevent phase smearing due to variation in individual μ-alpha frequency. Although, as expected, no oscillation is visible in random phase low-power trials, TMS was successfully delivered to peaks, falling flanks, troughs, and rising flanks in high-power trials. ***E***, Subjectwise average phase angles on the unitary circle and resulting mean vectors for estimated stimulation phase for each experimental condition and separately for single- (blue) and paired-pulse trials (red); due to TMS-related artifacts/potentials, stimulation phase was estimated for the μ-alpha cycle (see methods for details). The obvious phase offset of ∼18° between targeted and stimulated phase corresponds to ∼4.5 ms only, and is entirely due to technical delays (see Materials and Methods for details).

To ensure that TMS was correctly delivered to the intended μ-alpha states (Fig. 4) and that no systematic confounds occurred, we performed additional offline analyses of the pre-TMS time period with respect to power spectra (Fig. 4*A*), time-frequency representations (Fig. 4*B*), topographical distribution of μ-alpha power (Fig. 4*C*), time-locked EEG signal (Fig. 4*D*) and estimated phase of actual TMS delivery (Fig. 4*E*). These analyses revealed that on average power and phase were targeted as intended (with a technical delay of ∼4.5 ms, corresponding to ∼18° phase angle or 5% of the oscillatory cycle, see Materials and Methods), consistently across subjects (mean vector length across subjects was 0.96 for all phase targeted conditions and 0.15 for the random phase condition), and that neither adjacent frequencies nor oscillatory activity from other sources (such as occipital alpha) confounded the experimental variation of power and phase conditions.

## Discussion

We report evidence that the sensorimotor μ-alpha rhythm reflects asymmetric pulsed facilitation, rather than inhibition, of corticospinal excitability. Relative to a desynchronized, low power, μ-alpha state, MEP amplitudes were facilitated during high power troughs and rising flanks of the oscillation, but were not altered during peaks and falling flanks. Accordingly, we found no evidence for a link between GABA-A-receptor mediated intracortical inhibition and μ-alpha power or phase. These results bear immediate conceptual consequences. First, the observed pulsed facilitation of the motor cortex questions the universality of the pulsed inhibition hypothesis (Klimesch et al., 2007; Jensen and Mazaheri, 2010) beyond the realm of primary sensory regions. Second, the excitability difference between μ-alpha rising and falling flanks of comparable voltage amplitude challenges th*e function-through-biased-oscillations hypothesis* (Schalk, 2015), which assumes that instantaneous voltage amplitude, rather the power or phase of an oscillation reflects cortical excitability.

### μ-alpha rhythmically facilitates corticospinal excitability

μ-alpha troughs but not peaks were associated with facilitation of corticospinal excitability relative to periods of low μ-alpha power, but at no phase a relative inhibition could be observed. The resulting net facilitation of corticospinal excitability during the asymmetric μ-alpha oscillation corroborates recent findings of a weak positive relationship between μ-alpha power and MEP amplitude (Hussain et al., 2018; Thies et al., 2018; Ogata et al., 2019). While the larger excitability for troughs than peaks replicates previous findings (Schaworonkow et al., 2018, 2019; Stefanou et al., 2018; Zrenner et al., 2018), periods of spontaneous μ-alpha desynchronization (low power trials) had not yet been considered as baseline to determine the direction of phasic modulation. The only other study taking pre-TMS μ-alpha power into account used *post hoc* trial sorting of peaks and troughs and a trial-by-trial linear mixed-effects model to include continuous power values (Hussain et al., 2018). Notably, no main effect of phase but only an interaction with power was observed, driven by a positive relationship between μ-alpha power and MEPs during troughs but not peaks. If μ-alpha peaks simply reflect the absence of pulsed facilitation, but not active inhibition, as our results suggest, the amplitude of those peaks should indeed not matter, whereas the amplitude of troughs would reflect the degree of pulsed facilitation (Fig. 1*A*).

### μ-alpha does not modulate GABA-A-receptor mediated intracortical inhibition

Beside the lack of a phasic decrease in corticospinal excitability relative to desynchronized periods, there was also no evidence for a phasic modulation of GABA-A-receptor mediated inhibition as indexed by SICI (Kujirai et al., 1993; Di Lazzaro and Ziemann, 2013). SICI presumably reflects the feedforward inhibition of corticospinal cells via activation of inhibitory interneurons by the first subthreshold stimulus, as those interneurons likely have a lower excitation threshold (Di Lazzaro and Ziemann, 2013). Given constant excitability of corticospinal neurons, SICI should thus change whenever either the excitability of those inhibitory interneurons changes or the efficacy of their GABA-A-ergic transmission (Ilić et al., 2002). However, since corticospinal excitability was phasically modulated, comparable levels of relative SICI (% suppression of MEP) indicate variations in absolute inhibition (mV MEP amplitude). The excitability of both pyramidal cells and inhibitory interneurons thus seems proportionally facilitated during the μ-alpha trough, maintaining excitation–inhibition balance (EIB).

### Relevance of phase over instantaneous voltage amplitude

Despite similar absolute voltages at the zero crossings, corticospinal excitability was increased only during rising but not during falling flanks. Although the direct comparison between both flanks revealed a statistical trend only, these findings are not in support of the function-through-biased-oscillations hypothesis (Schalk, 2015), which argues that the absolute voltage and not phase per se explains phasic excitability changes. Our findings suggest that there is likely more to the oscillatory phase than absolute voltage. Because we exclusively used zero phase shift band-pass filters during real-time detection, and calculated *post hoc* time-locked averages from the unfiltered raw signal, it is unlikely that the observed flank asymmetry was spuriously produced by the asymmetric arch-like shape of the μ-rhythm (Cole and Voytek, 2017), which is characterized by different peak and trough duration but, to our knowledge, no particular asymmetry regarding its sharp rising and falling flanks. We can only speculate that the increase in corticospinal excitability during the rising flank may reflect a transient continuation of the neurophysiological process responsible for the facilitation during the trough itself.

### Potential mechanisms mediating μ-alpha related pulsed facilitation of corticospinal excitability

Given the predictions of the pulsed inhibition hypothesis (Klimesch et al., 2007; Jensen and Mazaheri, 2010), our results may appear controversial at first. However, in the primary somatosensory cortex (S1), the relationship between μ-alpha rhythm power and cortical excitability seems to be more complex than in the visual system (see Introduction), and there may be no uniform phase-excitability relationship within the sensorimotor system. The origin of the sensorimotor μ-alpha rhythm is presumably rather postcentral (S1), as opposed to the more precentral (M1) sensorimotor μ-beta rhythm (Salmelin and Hari, 1994; Ritter et al., 2009; Stolk et al., 2019), and Stolk et al. (2019) have recently demonstrated in ECoG recordings that the two rhythms are driven by different neuronal populations and are functionally segregated during movement selection. They even found that individual waves travel in opposite direction across the sensorimotor cortex, with alpha waves traveling from S1 to M1 and beta waves from M1 to S1 (Stolk et al., 2019). These traveling μ-alpha waves may in fact explain the considerable posterior-to-anterior μ-alpha phase shifts that are sometimes observable in the surface EEG, complicating the optimization of spatial filters for target signal extraction (Schaworonkow et al., 2018). Since the C3-Hjorth montage we used is likely more sensitive to radial sources from the crown of the postcentral gyrus (S1) than tangential sources from the anterior wall of the precentral sulcus (M1), our μ-alpha target signal may originate from a different neuronal population (in S1) than the one whose excitability we probed with MEPs (in M1). Given that the tight sensory-to-motor interconnections involve large amounts of feedforward inhibition (Murray and Keller, 2011), it is possible that μ-alpha causes pulsed inhibition in S1 (as predicted by the pulsed inhibition hypothesis) but a rhythmic release of M1 from a general sensory-to-motor inhibition. Future studies should explicitly investigate the role of S1-M1 interactions for μ-alpha power and phase effects.

It is also possible that the μ-alpha related pulsed facilitation of corticospinal excitability observed in this experiment only holds for the case of spontaneous μ-alpha oscillations at rest, whereas relative inhibition may be observable in MEP and SICI during μ-alpha de- and resynchronization in the context of motor tasks. Interestingly, such a state-dependent flip of effect direction has also been observed for TACS of the motor cortex at beta frequency (for a recent meta-analysis see Wischnewski et al., 2019), which paradoxically increased corticospinal excitability during rest (Feurra et al., 2011, 2013) but not during motor imagery (Feurra et al., 2013), while having the expected inhibitory or akinetic effect on motor performance (Pogosyan et al., 2009; Joundi et al., 2012). Then again, TACS at alpha frequency facilitated corticospinal excitability when applied during motor imagery rather than rest (Feurra et al., 2013). It has also been argued that beta-TACS induced synchronization of the relevant neuron populations in M1 may facilitate the recruitment of corticospinal neurons by the TMS pulse, synchronize the respective corticospinal volleys, and thereby increase MEP amplitude (Feurra et al., 2011). It is principally possible that also cortical synchronization by spontaneous alpha oscillations facilitates MEP amplitude via a similar mechanism.

Sensorimotor μ-alpha and μ-beta rhythms are physiologically and functionally separate rhythms that fluctuate independently (McFarland et al., 2000; Fransen et al., 2016; Stolk et al., 2019), and whereas beta was not investigated during this μ-alpha focused investigation, its precentral origin and clear motor task-related modulation make it a strong candidate for exerting power- and phase-specific effects on corticospinal excitability. However, in our previous studies we could not identify any such effects of beta by means of *post hoc* analyses, neither with respect to phase (Zrenner et al., 2018) nor power (Thies et al., 2018), and real-time beta-triggered TMS may be needed to answer that question in the future. Interestingly, Stolk et al. (2019) found the 1/f slope in the power spectrum, a putative power-spectral index of synaptic EIB (Gao et al., 2017), to indicate effector-specific and spatially focal shifts in EIB toward excitation during μ-beta power decreases in a motor imagery task, whereas the link between μ-alpha power and inhibition was spatially unspecific. A (potentially task-specific) dissociation between the EIB profile of μ-alpha and -beta oscillations, and their putative impact on corticospinal excitability, as indexed by the MEP, warrants future investigation.

### Conflicting evidence regarding the impact of μ-alpha power and phase on corticospinal excitability

Previous studies have either revealed no relationship of μ-alpha power with corticospinal excitability (Lepage et al., 2008; Berger et al., 2014; Keil et al., 2014; Schulz et al., 2014; Iscan et al., 2016; Madsen et al., 2019), a negative relationship for near-threshold stimulation intensities in very small samples (Zarkowski et al., 2006; Sauseng et al., 2009), or, more recently, a weak positive relationship (Hussain et al., 2018; Thies et al., 2018; Ogata et al., 2019). The impact of μ-alpha phase on corticospinal excitability was larger during troughs than peaks in all studies from our group (Schaworonkow et al., 2018; Stefanou et al., 2018; Zrenner et al., 2018; Schaworonkow et al., 2019), while one recent real-time EEG-triggered TMS study from another group did not observe this phasic modulation (Madsen et al., 2019). It should be noted that the samples from the above cited studies by our group (each with a different research question) partially overlapped. Of the total of *n* = 53 subjects, 31 subjects participated in a single study, 10 subjects in two studies, 8 subjects in three studies, and 4 subjects in four studies. For the current study, 7 subjects did not participate in any of the other studies, whereas 16 subjects also participated (before or afterward) in one or more of the other studies. Importantly, subjects were only included for their good μ-alpha peak in the power spectrum, while we were completely blind with respect to their individual expression of a phase effect. Madsen et al. argued that several previous studies also failed to find a μ-alpha phasic modulation of MEP amplitude (cf. their Table 1). However, the cited studies investigated corticomuscular coherence (van Elswijk et al., 2010; Keil et al., 2014; Schulz et al., 2014) or prestimulus power (Iscan et al., 2016), rather than μ-alpha phase; and van Elswijk et al. (2010) found phase effects in EMG (though not EEG) even during isotonic contraction, and Berger et al. (2014) reported EEG-MEP phase-amplitude correlations also in the μ-alpha range during rest. There are several issues, already mentioned by Madsen et al., that may be relevant for obtaining the observed phase effects. First, we preselected subjects based on the presence of a distinct μ-alpha peak in the C3-Hjorth power spectrum (here ∼64% of the screened subjects were included, but, importantly, no subject was removed thereafter). While such an inclusion criterion may reduce generalizability, it is necessary to ensure the correct implementation of the independent variable (i.e., μ-alpha phase). Without the presence of a clear oscillation, the most perfect detection algorithm will accurately target the meaningless phase of band-pass filtered 1/f noise (even for the upper percentiles of individual power values). Second, we consistently used C3-Hjorth montages, whereas Madsen et al. projected a dipole with radial orientation from the assumed cortical motor hot spot, potentially resulting in stronger contribution of more anterior sources (cf. their Fig. 2). Thirdly, their ITIs were much longer (mean 11.9 s ≈ 0.08 Hz) than ours (here: mean ± SD, 4.33 ± 1.08 s ≈ 0.23 Hz), and the large MEPs observed after particularly long ITIs (Julkunen et al., 2012) may have occluded the phase effect. Importantly, the irregular stimulation at ∼0.23 Hz has unlikely produced an “inhibitory brain state” (Madsen et al., 2019), and our stratification approach ensured equal ITIs across all phase conditions.

### Conclusion

Our findings are best explained by a scenario of pulsed facilitation of corticospinal excitability by power and phase of the sensorimotor μ-alpha rhythm, thus questioning whether the pulsed inhibition hypothesis (Klimesch et al., 2007; Jensen and Mazaheri, 2010) generalizes to the sensorimotor cortex and challenging the function-through-biased-oscillations hypothesis (Schalk, 2015). Future studies should test whether the observed pulsed facilitation actually relies on a rhythmic release from default sensory-to-motor inhibition.
